# Dimethyl Sulfoxide Induces Both Direct and Indirect Tau Hyperphosphorylation

**DOI:** 10.1371/journal.pone.0040020

**Published:** 2012-06-29

**Authors:** Carl Julien, François Marcouiller, Alexis Bretteville, Noura B. El Khoury, Joanie Baillargeon, Sébastien S. Hébert, Emmanuel Planel

**Affiliations:** 1 Département de Neurosciences et Psychiatrie, Faculté de Médecine, Université Laval, Québec, Québec, Canada; 2 Axe Neurosciences, Centre de Recherche du Centre Hospitalier Universitaire de Québec, Québec, Québec, Canada; Alexander Flemming Biomedical Sciences Research Center, Greece

## Abstract

Dimethyl sulfoxide (DMSO) is widely used as a solvent or vehicle for biological studies, and for treatment of specific disorders, including traumatic brain injury and several forms of amyloidosis. As Alzheimer’s disease (AD) brains are characterized by deposits of β-amyloid peptides, it has been suggested that DMSO could be used as a treatment for this devastating disease. AD brains are also characterized by aggregates of hyperphosphorylated tau protein, but the effect of DMSO on tau phosphorylation is unknown. We thus investigated the impact of DMSO on tau phosphorylation *in vitro* and *in vivo*. One hour following intraperitoneal administration of 1 or 2 ml/kg DMSO in mice, no change was observed in tau phosphorylation. However, at 4 ml/kg, tau was hyperphosphorylated at AT8 (Ser^202^/Thr^205^), PHF-1 (Ser^396^/Ser^404^) and AT180 (Thr^231^) epitopes. At this dose, we also noticed that the animals were hypothermic. When the mice were maintained normothermic, the effect of 4 ml/kg DMSO on tau hyperphosphorylation was prevented. On the other hand, in SH-SY5Y cells, 0.1% DMSO induced tau hyperphosphorylation at AT8 and AT180 phosphoepitopes in normothermic conditions. Globally, these findings demonstrate that DMSO can induce tau hyperphosphorylation indirectly *via* hypothermia *in vivo*, and directly *in vitro*. These data should caution researchers working with DMSO as it can induce artifactual results both *in vivo* and *in vitro*.

## Introduction

Dimethyl sulfoxide (DMSO), a polar aprotic solvent that dissolves a great variety of both polar and nonpolar substances, is miscible in many organic solvents as well as water and has been used for diverse laboratory and clinical purposes [1], [2]. DMSO can also be used as an additive, which improves absorbability of poorly water-soluble drugs without affecting the membrane integrity [3]. The compound has widespread pharmacological and research applications [2]. For instance, it is used as a drug carrier to cells [4] and as a cryoprotector [5], [6]. An example of the use of DMSO as a drug carrier in the Alzheimer’s disease (AD) field is the work on selective inhibitors of Glycogen Synthase Kinase-3 β (GSK-3β) *in vivo* and *in vivo* [7]–[10].

In clinical practice, DMSO can be used for treatment of specific disorders, including several forms of amyloidosis [2], but also in cardiac, or central nervous system damage such as traumatic brain injury (TBI) [11]. As AD brains are characterized by extracellular deposits of β-amyloid peptides, it has been suggested that DMSO could be used as a treatment for this devastating disease [2], [12]. AD and TBI brains are also characterized by intracellular aggregates of hyperphosphorylated tau protein, but the effect of DMSO on tau phosphorylation is unknown. Since DMSO could be a drug candidate in AD and TBI and since DMSO is widely used in the laboratory, notably as drug carrier for tau kinases inhibitors, we investigated the effect of DMSO on the phosphorylation of tau *in vivo* and *in vitro*.

One hour following intraperitoneal administration of 4 ml/kg DMSO in mice, but not at lower doses, tau was hyperphosphorylated at AT8 (Ser^202^/Thr^205^), PHF-1 (Ser^396^/Ser^404^) and AT180 (Thr^231^) epitopes. However, the mice were hypothermic at this dose, and maintaining them normothermic prevented the effect of DMSO. In SH-SY5Y cells, 0.1% DMSO induced tau hyperphosphorylation at AT8 and AT180 phosphoepitopes in normothermic conditions.

Globally, these findings demonstrate that DMSO can induce tau hyperphosphorylation indirectly *via* hypothermia *in vivo*, and directly *in vitro*.

## Results

### Tau Phosphorylation 1 h following the Administration of DMSO at 0, 1, 2, and 4 ml/kg i.p

**Figure 1:**
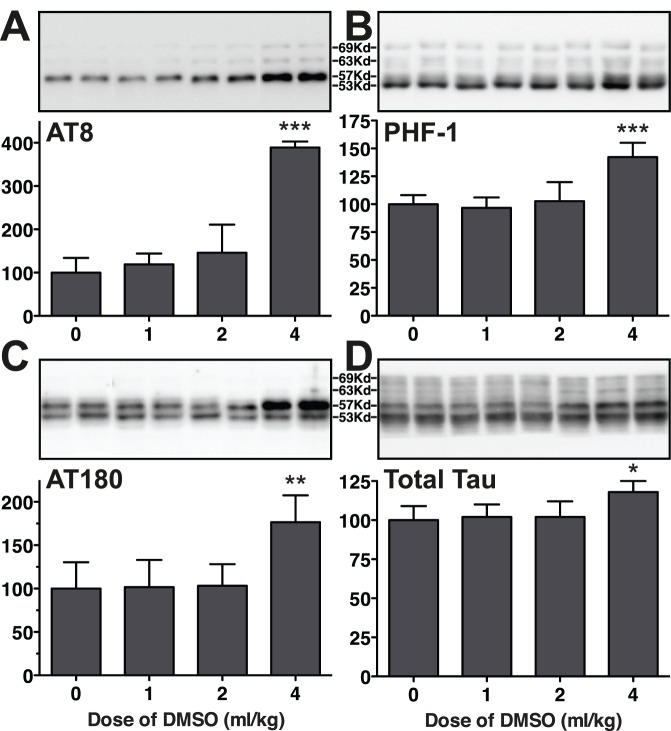
Tau phosphorylation in mouse hippocampal tissue 1 h following the administration of DMSO at 0, 1, 2, and 4 ml/kg i.p. Hippocampal protein extracts were separated by SDS-PAGE and levels of tau phosphorylation were determined using antibodies directed at the (A) AT8 (pSer^202^/pThr^205^), (B) PHF-1 (pSer^396^/pSer^404^), (C) AT180 (pThr^231^/pSer^235^) phophoepitopes, or (D) total Tau. For each phosphoepitope, relative immunoreactive band intensities are expressed as a ratio to total tau and are displayed as a percentage of control (0 ml/kg of DMSO). For each condition, 2 representative data are displayed with controls (0 ml/kg; n  = 5), 1 ml/kg (n  = 5), 2 ml/kg (n  = 5) and 4 ml/kg (n  = 5). Data are expressed as mean ± SD. *, **, *** denote *p*<0.05, *p*<0.01 and *p*<0.001 versus control, respectively; ANOVA with Newman-Keuls *post hoc* test.

We first examined whether DMSO i.p. doses lead to a change in tau phosphorylation in mice. For this purpose, western blot analyses with antibodies targeting key tau phosphoepitopes were used in hippocampus homogenate from mouse injected with either 0, 1, 2 or 4 ml/kg DMSO i.p. These doses are well below the reported LD50 of 13.4–15.5 g/kg (12.2–14.1 ml/kg) in mice [13], [14], and indeed, we did not detect cell death or induction of apoptosis as determined by examining Poly (ADP-ribose) polymerase cleavage or caspase 3 activation (data not shown). AT8, PHF-1 and AT180 antibodies are specific to tau phosphorylated at Ser^202^/Thr^205^, Ser^396^/Ser^404^, and Thr^231^/Ser^235^, respectively, which are known to be highly phosphorylated during tau pathogenesis [15]–[19]. One hour following administration, DMSO produced a significant increase in hippocampal tau phosphorylation (expressed as a ratio of phosphotau to total tau) at the AT8 (+289%), PHF-1 (+42%), and AT180 (+76%) phosphoepitopes in mice injected with an i.p. dose of 4 ml/kg, when compared to the control group (0 ml/kg) (Fig. 1A-C). One or 2 ml/kg did not lead to change in tau phosphorylation (Fig. 1A-C). A slight increase in total tau in the 4 ml/kg group was also observed (+18%) compared to the control (0 ml/kg) (Fig. 1D). We have previously observed slight increases in total tau following hypothermia induced by anesthesia [20] or diabetes [21], but we do not know the cause of this phenomenon at this time. Nevertheless, our results demonstrate that DMSO can induce tau hyperphosphorylation at multiple epitopes in the mouse brain.

### Immunohistochemistry also Reveals a Tau Hyperphosphorylation Effect of DMSO

**Figure 2:**
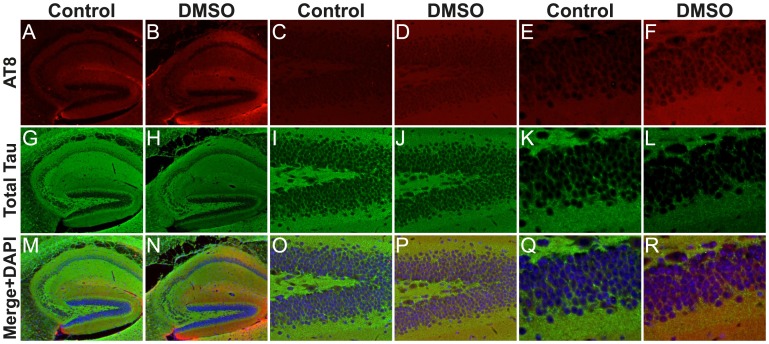
Regional anatomical localization of phosphorylated tau protein in DMSO treated mice at 5X (A-B, G-H, M-N), 20X (C-D, I-J, O-P) and 40X magnification (E-F, K-L, Q-R). Fluorescent photomicrographs of hippocampal sagittal sections are shown with AT8 (Red, A-F), Total Tau (Green, G-L), or merged with DAPI (M-R), for the following groups: control (A, C, E, G, I, K, M, O, Q) or DMSO-treated mice (B, D, F, H, J, L, N, P, R).

Immunostaining of hippocampal sagittal sections of mice revealed a robust hyperphosphorylation of tau at the AT8 epitope 1 h after injection of the highest dose of DMSO (Fig. 2B, D, F) compared to controls (Fig. 2A, C, E). The immunohistochemistry results are in accordance with our Western blot data (Fig. 1A). In AD, the hyperphosphorylation of tau occurs first in neurites and is then followed by somatodendritic relocalization [22]. Here, no obvious somatodendritic relocalization was observed in our immunostainings. These results confirm our Western blot analysis and show that DMSO did not induce a shift of tau from the axons to the soma.

### Impact of DMSO on Tau Kinases

**Figure 3:**
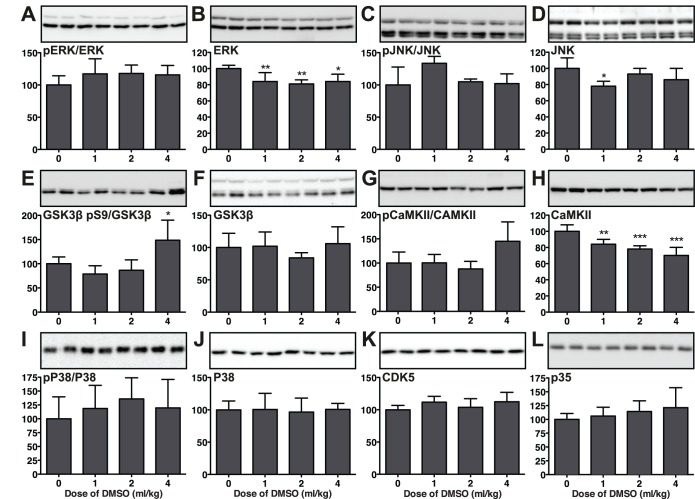
Effect of DMSO on tau kinases in mouse hippocampal tissue 1 h following the administration of DMSO (4 ml/kg i.p.). Hippocampal protein extracts were separated by SDS-PAGE and levels of kinases were determined using antibodies directed at activated or total kinases as follow: (A) phospho-ERK/total ERK ratio, (β) total ERK, (C) phospho-JNK/total JNK ratio, (D) total JNK, (E) GSK-3β phospho-S9/total GSK-3β ratio, (F) total GSK-3β, (G) phospho-CaMKII/total CaMKII ratio, (H) total CaMKII, (I) phospho-P38/total P38 ratio, (J) total P38, (K) CDK5, and (L) p35. Relative immunoreactive band intensities are expressed as a percent of control (0 ml/kg of DMSO) and are displayed for each epitope. For each condition, 2 representative data are displayed with controls (0 ml/kg; n  = 5), 1 ml/kg (n  = 5), 2 ml/kg (n  = 5) and 4 ml/kg (n  = 5). Data are expressed as mean ± SD. *, **, *** denote *p*<0.05, *p*<0.01, *p*<0.001 versus control; ANOVA with Newman-Keuls *post hoc* test.

We next attempted to clarify the mechanism of DMSO-induced hyperphosphorylation by examining the activation of specific tau kinases commonly involved in the regulation of tau phosphorylation 1 hr following 1, 2, and 4 ml/kg of DMSO i.p. We explored the activation patterns of seven kinases with specific antibodies in the same hippocampal tissues previously used for tau phosphorylation analysis. A dose of 4 ml/kg of DMSO significantly increased phospho-GSK-3β (Ser9, inhibitory phosphorylation) levels (+58%; Fig. 3E), but pERK/ERK ratio (Fig. 3A), pJNK/JNK ratio (Fig. 3C), pCaMKII/CaMKII ratio (Fig. 3G), phospho-P38 MAPK/P38 MAPK ratio (Fig. 3I), CDK5 (Fig. 3K) and p35 (Fig. 3L) did not change compared to controls (0 ml/kg). Our results fail to explain the increase in tau phosphorylation in terms of kinases activation.

### Effect of DMSO on Tau Phosphatases

**Figure 4:**
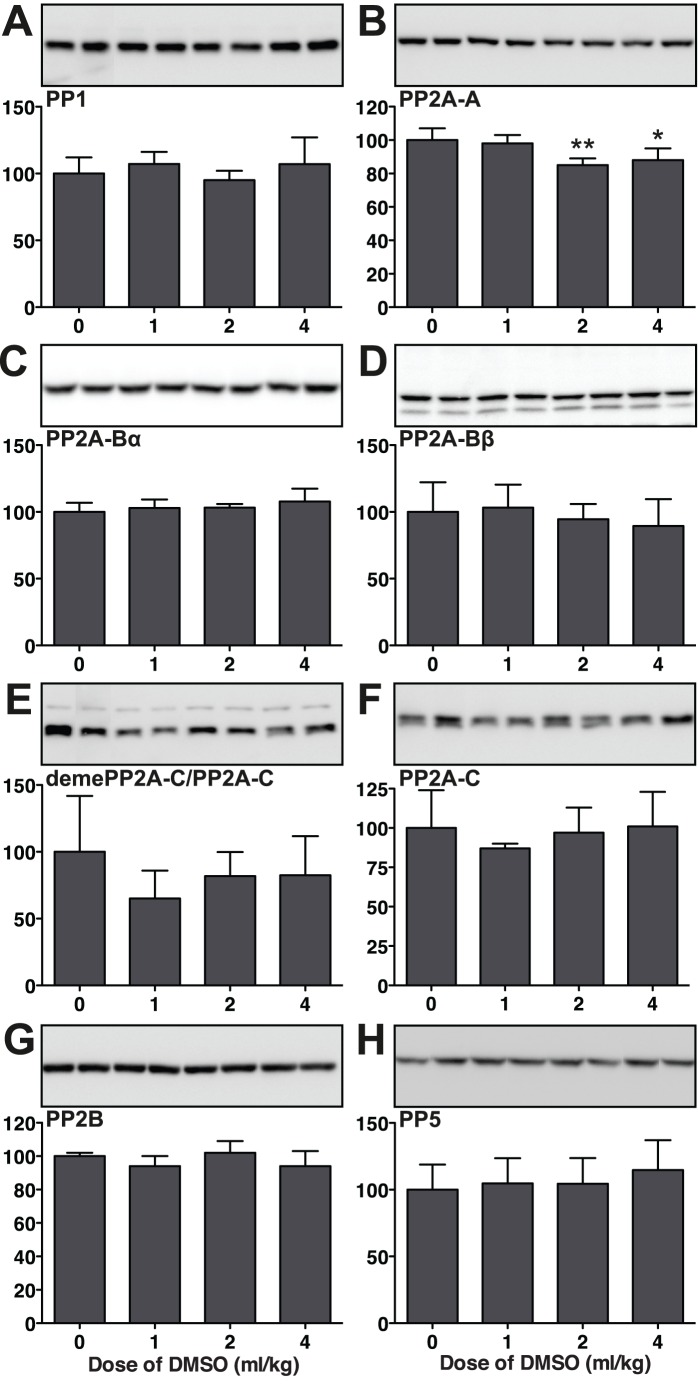
Effect of DMSO on tau phosphatases in mouse hippocampal tissue 1 h following the administration of DMSO (4 ml/kg i.p.). Hippocampal protein extracts were separated by SDS-PAGE and levels of phosphatases were determined using antibodies directed at activated or total kinases as follows: (A) PP1, (B) PP2A regulatory subunit A, (C) PP2A-Bα subunit, (D) PP2A-Bβ subunit, (E) demethylated PP2A catalytic C subunit/total PP2A catalytic C subunit ratio, (F) total PP2A catalytic C subunit, (G) PP2B, and (H) PP5. Relative immunoreactive band intensities are expressed as a percent of control (0 ml/kg) and are displayed for each epitope. For each condition, 2 representative data are displayed with controls (0 ml/kg; n  = 5), 1 ml/kg (n  = 5), 2 ml/kg (n  = 5) and 4 ml/kg (n  = 5). Data are expressed as mean ± SD. *, ** denote *p*<0.05, *p*<0.01 versus control, respectively; ANOVA with Newman-Keuls *post hoc* test.

As no changes in kinases could explain the hyperphosphorylation of tau AT8, PHF-1 and AT180 phosphoepitopes, we examined next the levels of the catalytic subunits of PP1, PP2B, PP2A, and PP5 1 h after DMSO administration. Tau can be dephosphorylated by PP1, PP2A, PP2B, and PP5, but PP2A shows a much stronger ability to dephosphorylate tau [23], [24]. Immunoblot analysis revealed a slight decrease of the regulatory subunit A of PP2A 1****h after administration of 2 (-15%) or 4 ml/kg (-12%) of DMSO (Fig. 4B). As PP2A A is decreased at both 2 and 4 ml/kg, it is unlikely to be the cause of tau hyperphosphorylation at the higher dose. There was no change in PP1, PP2A-Bα and PP2A-Bβ subunits, demethylated PP2A-C/PP2A-C ratio, PP2B, or PP5 levels was observed following 1, 2, or 4 ml/kg of DMSO i.p., compared to controls (0 ml/kg) (Fig. 4A, C-H). Here again, there was no clear correlation between tau hyperphosphorylation and changes in phosphatases levels.

### Four ml/kg DMSO Induces a Transient Hypothermia After 1****h in Mouse

**Figure 5:**
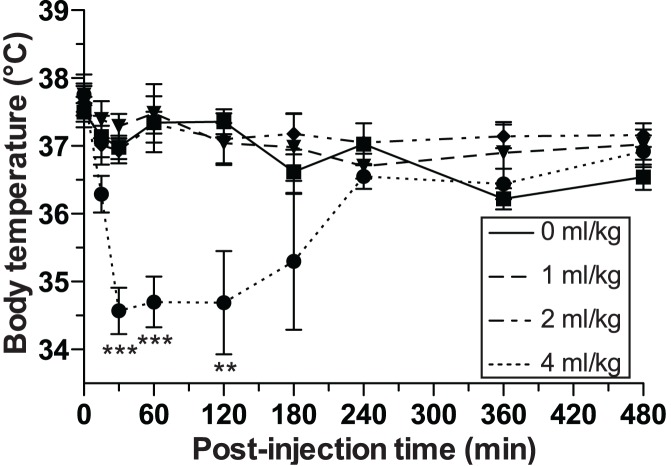
Body temperature follow-up following 0 (n  = 4–22), 1 (n  = 4–10), 2 (n  = 4–10) and 4 ml/kg (n  = 4–28) of DMSO i.p administration. Data are expressed as mean ± SEM. **, *** denote *p*<0.01, *p*<0.001 versus control (0 ml/kg of DMSO); ANOVA with Newman-Keuls *post hoc* for each time point.

Since tau hyperphosphorylation may be the consequence of hypothermia [20], [25]–[31], we examined whether DMSO i.p. doses led to a change in body temperature in mouse. Four ml/kg of i.p. DMSO decreased the mouse rectal temperature to 34.6±0.3°C versus 37.0±0.2°C 30 min after administration (*p*<0.001), 34.7±0.4 versus 37.3±0.2°C 60 min after administration (*p*<0.001), and 34.7±0.8°C versus 37.4±0.2°C 120 min after administration (*p*<0.001) from the control group (0 ml/kg) (Fig. 5). However, i.p. doses of 1 and 2 ml/kg of DMSO did not significantly change the body temperature of mice at any time point (Fig. 5). Thus, tau hyperphosphorylation after injection of DMSO seems to be associated with hypothermia at 4 ml/kg.

### Normothermia Prevents DMSO-induced Tau Hyperphosphorylation

**Figure 6:**
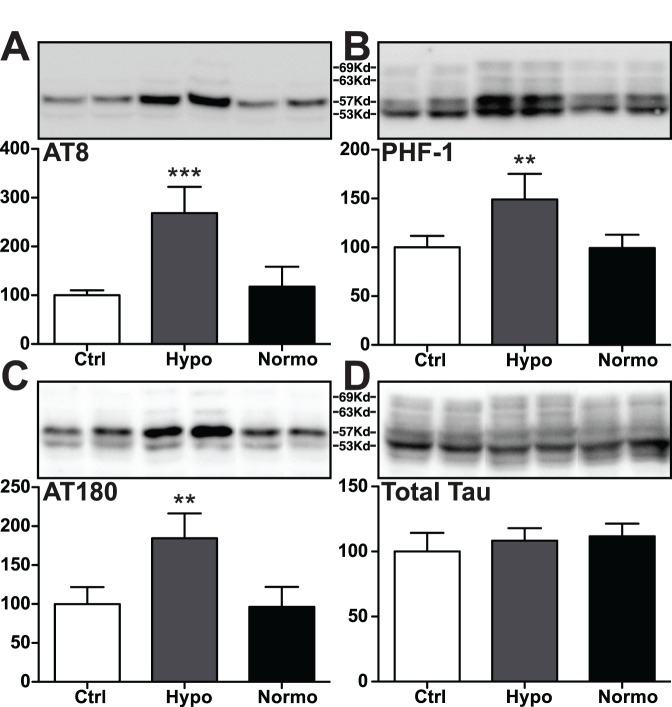
Tau phosphorylation in mouse hippocampal tissue 1 h following the administration of DMSO (4 ml/kg i.p.) under hypothermic (Hypo) and normothermic (Normo) conditions. Hippocampal protein extracts were separated by SDS-PAGE and levels of tau phosphorylation were determined using antibodies directed at the (A) AT8 (pSer^202^/pThr^205^), (B) PHF-1 (pSer^396^/pSer^404^), (C) AT180 (pThr^231^/pSer^235^) phophoepitopes, or (D) total Tau. For each phosphoepitope, relative immunoreactive band intensities are expressed as a ratio to total tau and are displayed as a percentage of control (0 ml/kg of DMSO). For each condition, 2 representative data are displayed with controls (0 ml/kg; n  = 5), 4 ml/kg DMSO in hypothermic conditions (n  = 5), and 4 ml/kg DMSO in normothermic conditions (n  = 6). Data are expressed as mean ± SD. **, *** denote *p*<0.01, *p*<0.001 versus control, respectively; ANOVA with Newman-Keuls *post hoc* test.

To demonstrate a causative effect of hypothermia on tau hyperphosphorylation at 4 ml/kg DMSO, the body temperature of a group of DMSO-injected mice was maintained at ∼37°C using a ventilated incubator. In the hypothermic group, 1 hr following administration, 4 ml/kg DMSO produced a significant increase in hippocampal tau phosphorylation at the AT8 (+168%), PHF-1 (+49%), and AT180 (+84%) phosphoepitopes in mice, when compared to the control group (0 ml/kg; Fig. 6A-C), in accordance with our dose-response analysis (Fig. 1). However, tau hyperphosphorylation at the AT8, PHF-1 and AT180 epitopes was prevented in the normothermic group (Fig. 6A-C). Levels of total tau remained unchanged in hypothermic and in normothermic conditions compared to controls (0 ml/kg; Fig. 6D). Therefore, hypothermia seems to be the indirect cause of the effect of 4 ml/kg DMSO on tau hyperphosphorylation at multiple epitopes in the mouse brain.

### DMSO Induces Tau Hyperphosphorylation in SH-SY5Y Cells

**Figure 7:**
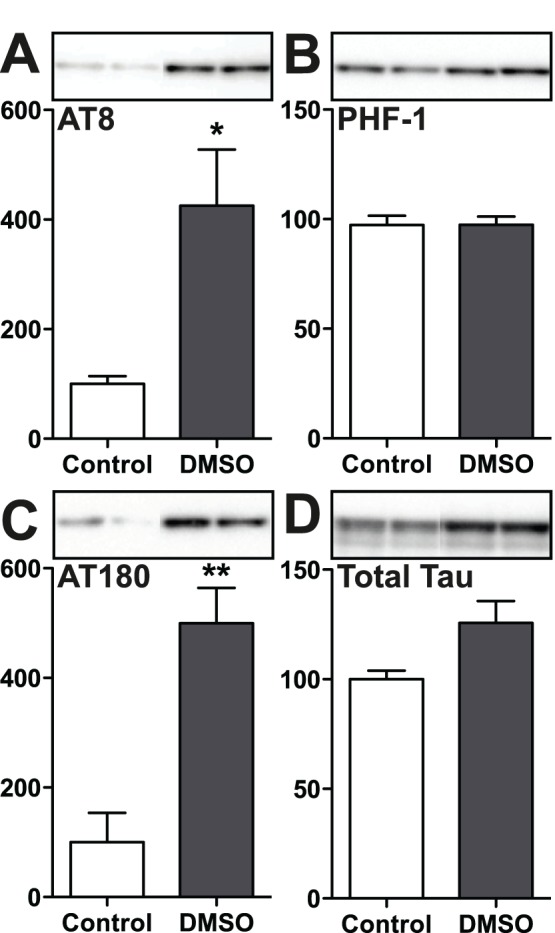
Effect of DMSO on tau phosphorylation in 3R human tau transfected SH-SY5Y cells following 1 h exposure. Cell lysate protein extracts were separated by SDS-PAGE and levels of tau phosphorylation were determined using antibodies directed at the (A) AT8, (B) PHF-1, or (C) AT180 phosphoepitopes, and (D) total tau. Relative immunoreactive band intensities are expressed as a percent of control from a total Tau ratio and are displayed for each phosphoepitope and total tau. For each condition, 2 representative data are displayed with control (n  = 9) and 0.1% DMSO (n  = 9). Data are expressed as mean ± SD. *, ** denote *p*<0.05 and *p*<0.01, versus control, respectively; Student’s t test.

As we have seen above, one of the effects of DMSO is indirect, via hypothermia at a 4 ml/kg dose. But we also wanted to explore the effects of direct application of DMSO on neuronal-like cells. Thus, we next determined the effect of DMSO on tau phosphorylation at the AT8, AT180 and PHF-1 phosphoepitopes using SH-SY5Y human neuroblastoma cells stably transfected to express human tau with 3 microtubule binding domains (htau SH-SY5Y cells). We choose to expose the cells to 0.1% DMSO because it is roughly equivalent to the concentration of DMSO in the brain after 1****h following injection of 4 ml/kg i.p. [32]. One-hour exposure to 0.1% DMSO increased tau phosphorylation at AT8 (+325%) and AT180 (+400%) phosphoepitopes (Fig. 7A, C). Phosphoepitope PHF-1 and total tau remained unchanged after 0.1% DMSO exposure in htau SH-SY5Y cells (Fig. 7B, D). These results demonstrate that application of DMSO onto neuronal cells can lead to direct tau hyperphosphorylation.

## Discussion

The work presented here provides novel evidence that DMSO, which is extensively used in laboratory, increases tau phosphorylation indirectly *via* hypothermia *in vivo*, and directly *in vitro*.

We first examined the effect of increasing doses of DMSO from 1 to 4 ml/kg on tau phosphorylation in mice. *In vivo*, DMSO is used at a wide range of concentrations. For example, doses of 0.1 ml/kg [33], 1 ml/kg [34], 2 ml/kg [35], [36], and 4 ml/kg [37] or higher [38] have been utilized. Here, we observed an increase in tau phosphorylation at all the examined epitopes (AT8, AT180, PHF-1), only at 4 ml/kg. All these tau epitopes are highly phosphorylated in AD [15]–[19], and have been associated with paired helical filament and neurofibrillary tangles formation [39]. Therefore, it appears that DMSO exposure can result in an increase in tau phosphorylation at epitopes that are critical for the development of tau pathology.

We also noticed that the mice were hypothermic following a 4 ml/kg dose. DMSO has previously been documented to induce hypothermia in rats and mice [32], [40] but the minimal dose for this effect was not reported. Here we demonstrate that doses of 1 or 2 ml/kg did not induce hypothermia, but 4 ml/kg and above (6 ml/kg, data not shown) did in a 30 to 120 min timeframe following i.p. administration. We have previously demonstrated that tau phosphorylation *in vitro* or *in vivo* is exquisitely sensitive to hypothermia, and that hypothermia induced by a variety of drugs such as anesthetics or by alteration of metabolism will always result in hyperphosphorylation at multiple epitopes by direct inhibition of PP2A [20], [25]–[28]. Here, DMSO at 4 ml/kg induced hypothermia and resulted in an extensive tau hyperphosphorylation that was rescued by maintaining the animals normothermic. These results demonstrate that DMSO-induced tau hyperphosphorylation is indirect and due to hypothermia *in vivo*.

We also observed an increase in GSK-3β Ser9 phosphorylation at 4 ml/kg DMSO. This enhanced GSK-3β Ser9 phosphorylation is seen in different animal models undergoing hypothermia [20], [21], [26], [27], [31] and is probably mediated through the inhibition of phosphatases such as PP2A at low temperatures. Indeed, inhibition of PP2A by okadaic acid has been demonstrated to increase Ser9 phosphorylation [41], [42].

DMSO is widely used *in vitro* at 0.1% or less [9], [43], [44]. Some studies also reported the utilization of DMSO at 1% *in vitro* [45]. Exposure of 0.1% DMSO in Tau-SH-SY5Y human neuroblastoma cells increased tau phosphorylation at AT8 and AT180 phosphoepitopes, but not at PHF-1, demonstrating that DMSO can directly increase tau phosphorylation, albeit at specific epitopes. DMSO readily crosses the blood-brain barrier [46], so the lack of direct effect of DMSO *in vivo* compared to *in vitro* is probably not a problem of neurons being not exposed to DMSO. There are several possible explanations for this discrepancy. One is that tau in the mouse brain is mainly axonal, while it is cytosolic in the cells, thus being exposed to different populations of kinases and phosphatases. Another explanation could be that neurons *in situ* require higher doses of DMSO than SH-SY5Y cells to display tau hyperphosphorylation, as 0.1% of DMSO *in vitro* is roughly equivalent to the brain concentration of DMSO after an i.p. injection of a 4 ml/kg dose [32]. This hypothesis could be tested by injecting DMSO at higher doses in mice and maintain them normothermic. However, our animal ethic committee has not allowed us to utilize DMSO above 4 ml/kg i.p. in mice, except to obtain pilot data at the beginning of the study.

Globally, our data demonstrate that DMSO can induce *in vitro* direct, and *in vivo* indirect (*via* hypothermia) hyperphosphorylation of tau. Our results should caution researchers working with DMSO and examining tau protein both *in vitro* and *in vivo*. When working with animals, we recommend to always check their temperature, and to maintain them normothermic when using doses of 4 ml/kg or above.

## Materials and Methods

### Animals

The study and the use of animals were approved by the Comité de Protection des Animaux du CHUL and all procedures were performed according to the guidelines of the Canadian Council on Animal Care. Four month-old C57/129 mice of either sex were utilized in this study. The mice were housed in a temperature-controlled room at 22°C and were kept on a 12****h/12****h light/dark cycle. All animals had access to food and water *ad libitum*.

### DMSO Exposure

Unless otherwise noted, reagents were obtained from Sigma-Aldrich (St. Louis, MO). Animals were exposed to 0, 1, 2 or 4 ml/kg of 99.7% pure DMSO (#BP231, Fisher Scientific, Ottawa, ON, Canada) via intraperitoneal (i.p.) injections from a 10% solution of DMSO in 0.9% NaCl freshly prepared. An equal volume of 0.9% NaCl was injected in the controls. In hypothermic conditions, mice were kept at room temperature. In normothermic conditions, the body temperature was maintained at ∼37°C in a ventilated incubator. Rectal temperatures were monitored using an electronic thermometer probe (Thermalert TH-5, Physitemp, Clifton, NJ).

### Preparation of Mouse Brain Protein Extracts

Following DMSO exposure, mice were killed by decapitation without anesthesia, since anesthesia increases hypothermia-induced tau phosphorylation [26], 1****h following vehicle or DMSO administration. Brains were immediately removed and dissected on ice within one minute. The tissues were immediately frozen on dry ice and stored at -80°C. Hippocampal protein extracts were prepared by sonicating (MP Biomedicals, Solon, OH) the frozen hippocampi in 10X vol/wt RIPA buffer containing 50 mM Tris-HCl, pH 7.4, 1 mM EDTA, 150 mM NaCl, 0.5% sodium deoxycholate, 1% NP-40, phosphatase inhibitors (1 mM sodium vanadate and 1 mM sodium fluoride), and protease inhibitors (Proteases Inhibitors Cocktail P8340, Sigma-Aldrich, 10 µl/ml, and 1 mM PMSF). The samples were then centrifuged at 20,000****g for 20 min at 4°C, and the supernatants were recuperated and kept stored at -80°C.

### Cell Culture Studies and Cell Lysate Protein Extract Preparation

DMSO-induced tau phosphorylation was also examined using SH-SY5Y human neuroblastoma cells stably transfected to constitutively express human tau with 3 microtubule binding domains [39], [47]–[49]. These 3R tau SH-SY5Y cells were a kind gift of Dr. Luc Buée. The cells were maintained in DMEM with 10% fetal calf serum (Hyclone, Thermo Fisher Scientific, Waltham, MA, USA), 2 mM L-glutamine (Invitrogen, Carlsbad, CA) in a 5% CO_2_ humidified incubator at 37°C. In preparation for DMSO or vehicle exposure, the cells were transferred into dishes and used at approximately 70% confluency. On the day of the experiment, the growth medium was removed by aspiration, and each well was washed 2 times with 1 mL of fresh DMEM containing no additives. The cells were incubated with 0.1% DMSO or 1X PBS for the controls for 1 h at 37°C. Following the exposure, the DMSO or control solutions were removed by aspiration, and each plate was rinsed 2 times with 1 mL of PBS. The cells were harvested in ice-cold RIPA buffer containing phosphatase and protease inhibitors (same inhibitors as mouse brain protein extracts in previous section). The lysate was then centrifuged at 20,000 g for 20 min at 4°C, and the supernatant was collected and analyzed for protein content using a bicinchoninate acid (BCA) assay kit (Thermo Fisher scientific). Samples were then stored at −80°C until immunoblotting analyses.

### Western Blot Analysis

Samples were processed as previously described [25], with modifications. Briefly, proteins were mixed with NuPAGE® LDS Sample Buffer (Invitrogen) containing 5% 2-mercaptoethanol and boiled for 10 minutes. The expressions of phosphorylated tau, total tau as well as tau kinases and phosphatases were determined using SDS-polyacrylamide gel electrophoresis (PAGE) coupled with Western blot analysis. Brain homogenate or cell lysate extracts containing 20 µg protein were separated on a SDS-10% polyacrylamide gel and then transferred onto nitrocellulose membranes (Amersham Biosciences, Pittsburgh, PA). Non-specific binding sites were blocked with 5% nonfat dry milk in Tris-buffered saline containing 0.1% Tween 20 for 1 h at room temperature at then were incubated overnight at 4°C with antibodies directed against either total tau, phosphorylated tau, specific tau kinase or phosphatase. The following day, membranes were washed 3 times and then incubated for 1 h at room temperature with a horseradish peroxidase-linked secondary anti-mouse or anti-rabbit antibody (Cell Signaling Technology, Danvers, MA, 1∶5,000 dilution), and the immunoreactive band signal intensity was visualized by enhanced chemiluminescence (ECL Plus, GE Healthcare Biosciences, Piscataway, NJ). The immunoreactive bands were scanned using Fujifilm LAS 4000 imaging system (GE Healthcare Biosciences, Piscataway, NJ) and densitometric analysis was performed on these scans with ImageGauge analysis software (Fujifilm USA, Valhalla, NY).

### Antibodies

Monoclonal antibodies were utilized in the study that were directed at tau phosphorylated at the following epitopes: AT8 (pSer^202^/pThr^205^, MN1020, [16] 1∶1000 dilution, Thermo Scientific, Rockford, IL), AT180 (pThr^231^, 1∶1000 dilution, Thermo Scientific), PHF-1 (pSer^396^/Ser^404^ [50], 1∶1000). Total tau was detected using Tau A0024 (polyclonal, 1∶1000, Dako Cytomation, Carpinteria, CA, USA) which detects all 6 six isoforms of tau. The PHF-1 antibody was a generous gift from Dr. Peter Davies (Albert Einstein University, New York, NY).

Changes in tau kinases were examined using GSK-3β (1∶1000, BD Transduction Lab, Franklin Lakes, NJ), and the following antibodies purchased from Cell Signaling: phospho-GSK-3β (Ser^9^, 1∶1000), SAPK/JNK (1∶1000), phospho-SAPK-JNK (Thr^183^/Tyr^185^, 1∶1000), p44/42 MAPK (Erk 1/2, 1∶1000), phospho p44/42 MAPK (Erk 1/2, Thr202/Tyr204, 1∶1000). Cyclin dependent kinase 5 (Cdk5, C-8, 1∶1000), p35 (C-19, 1∶1000) and phospho-Ca^2+^/calmodulin-dependent protein kinases II (CAMKII, Thr^286^, 1∶1000) and total CamKII (1∶1000) were purchased from Santa Cruz Biotechnology, Santa Cruz, CA, and p38 MAP Kinase (1∶1000) and Phospho-p38 MAPK (Thr^180^/Tyr^182^, 1∶1000) were purchased from New England BioLabs (Ipswich, MA).

Changes in tau phosphatases were examined using PP1 catalytic subunit (E-9, 1∶1000, Santa Cruz), PP2A-A subunit (1∶1000, Cell Signaling), PP2A-Bα subunit (PPP2R2A (2G9), 1∶1000, Cell Signaling), PP2A-Bβ subunit (PPP2R2B, 1∶1000, Bethyl Laboratories, Montgomery, TX), PP2A-C subunit (1∶1000, Cell Signaling), demethylated PP2A-C subunit (1∶1000, Millipore, Billerica, MA), pan-calcineurin A (PP2B, 1∶1000, Cell Signaling), and PP5 (1∶1000, Cell Signaling).

### Immunofluorescence

Tissue fixation was done according to the “cold Bouin’s method” [27]. Briefly, animals were killed by decapitation without anesthesia to avoid tau hyperphosphorylation [26], the brain was quickly removed and immersed in ice-cold Bouin’s solution (saturated picric acid, formalin, acetic acid at 15∶5:1, v/v/v) for 24 hr and embedded in paraffin blocks. Eight to ten micrometer thick sections were processed for immunohistochemical analyses. Deparaffinized and hydrated sections were incubated in Target Retrieval Solution (Dako, Carpinteria, CA) at 70°C for 25 min for enhancement of the immunoreactivity and then incubated in 7% normal goat serum, 0.2% triton, 1% BSA in PBS 0.1 M at RT for 1 hr. The specimens were incubated with the primary antibodies diluted in PBS 0.1 M containing 0.04% triton overnight at 4°C. The following antibodies were used: AT8 (monoclonal, 1∶200 dilution, Pierce Biotechnology, Rockford, IL) and Tau A0024 (polyclonal, 1∶500, Dako Cytomation, Carpinteria, CA, USA). Bound antibodies were visualized with Alexa Fluor 568 conjugated anti-mouse IgGγ1 (1∶500 dilution) or Alexa Fluor 488 conjugated anti-rabbit IgG (1∶1000 dilution) (Molecular Probes, Eugene, OR). Slices were mounted with a Vectashield Hard Set Mounting Medium containing DAPI (Vector Laboratries, Inc, Burlingame, CA). Immunolabeled tissues were observed under a Carl Zeiss Axio Imager M2 microscope equipped with AxioCam MRm (Carl Zeiss, Jena, Germany) and a Nuance FX multispectral imaging system (Cambridge Research & Instrumentation, CRi, Woburn, MA) and a Nuance 2.10 software.

### Statistical Analysis

Group comparisons of immunoblot relative band intensities were performed using a one-way analysis of variance (ANOVA) with Newman-Keuls Multiple Comparison *post hoc* test applied when appropriate or by means of an unpaired *t*-test. Statistical calculations were performed using Prism 5 software (GraphPad Software, Inc., San Diego, CA) and JMP 8 (SAS Institute, Cary, NC), and all data are reported as mean ± SD or SEM, with a value of *p<*0.05 considered statistically significant.
